# Applying the HEART score is safe and saves

**DOI:** 10.1007/s12471-022-01710-x

**Published:** 2022-07-05

**Authors:** Y. Appelman, P. Doevendans

**Affiliations:** 1 Department of Cardiology, Amsterdam Cardiovascular Sciences, Amsterdam UMC, VU University, Amsterdam, The Netherlands; 2 Department of Cardiology, University Medical Centre Utrecht, Utrecht, The Netherlands; 3 Netherlands Heart Institute, Utrecht, The Netherlands

Koper et al. performed an encouraging study (Urgent 1.5) to fine-tune the well-established HEART score [1], which was previously initiated and validated by the cardiologist Jacob Six. He and several PhD students looked at various aspects of the score, which was based on clinical intuition and experience to rule out an acute coronary syndrome (ACS). However, not every parameter (history, electrocardiogram [ECG], age, risk factor and troponin) or value (0, 1, 2) used to generate the score was validated initially. Yet the score outperformed all competitors, also because of patient selection. Interesting was the finding that calculation of the C‑statistic to determine the weight of the 5 items times 3 values provided a 99% match [2]. The score was designed to rule out serious ACS in chest pain patients, who are swamping healthcare facilities. Therefore we focus on the low-risk group (HEART score 0–3) visiting the emergency department (ED) with potentially the largest gain for both patients and physicians. Even in low-risk patients many expensive diagnostic tests are performed which are time consuming and put the patient at risk during invasive procedures [3]. Even in the small study reported by Koper et al. [1], in patients with a low-risk score a very high sensitivity (97%) and negative predictive value (97.6%) are reported using the modified HEART score, and only one ACS was missed. Importantly, the study included a point-of-care (POC)-based test assessed by a fingertip troponin I measurement suitable for the home situation, which was, however, obtained at the ED. Although the modified HEART score including the early POC led to reclassification, the outcome was comparable to that using the original approach.

These data match the findings of earlier studies, indicating that it does not really matter which troponin test is used: POC, I/T or high-sensitivity troponin, although the score was initially tested using standard troponin I/T measurements [4].

There are several suggestions based on this study [1]. Importantly, this is further confirmation that in chest pain patients the HEART score is extremely useful in defining low-risk patients in whom a wait-and-see policy is very safe. Koper et al. reported just one (6%) ACS; the relatively high percentage is misleading, as it can be caused by the small study size (*n* = 96). Earlier studies reported 1.7% [5] and 0% [6]. In addition, if the fingertip POC troponin test is performed at home and the patient is not transported to the hospital or admitted, this will save time and resources as well as improve the quality of care.

Cost saving was studied earlier in a stepped wedge, randomised, cluster-based trial to introduce the HEART score in consecutive Dutch hospitals. Poldervaart et al. again showed the safety of the score used in chest pain patients admitted to the ED. The event rate was 2% in the low-risk group and lower when the HEART score was used compared to the usual care group, prior to introduction of the HEART score [7]. The study failed to show a marked cost reduction, as the low-risk group was often admitted despite the favourable prognosis. Also, the risk-avoiding behaviour of the attending physicians or other steering incentives may have obscured the saving potential.

The chest pain population in the current study is clearly different from ACS study patients. Most studies based on chest pain presentation at the ED show a 50% inclusion rate for women (Koper et al., 47%). Yet in most ACS studies this is between 20 and 30% depending on the inclusion criteria [8]. This higher inclusion rate of females in chest pain studies may have contributed to the findings of Bank et al., who showed increased safety in the retrospective analysis of the MINERVA study, which included almost 2000 patients. In low-risk female patients the event risk was 2.1 versus 6.5 in men [9].

The introduction of POC troponin I assessment does not reduce the validity of the HEART score. The POC test can be performed significantly earlier compared with hospital-based measurements. It is not clear how much delay will be introduced by waiting for the POC outcome and home assessment. Yet if the ECG does not prove an ACS, some investment of time at home will eventually benefit the chain of care. Also very early tests could still be negative despite the presence of an ACS. Therefore we concur with Koper et al. [1] that the next step should be a general practitioner and ambulance staff based study potentially using new home-based ECG technology together with the fingertip POC troponin assessment. Whether the same kit can be used for both sexes needs to be clarified, as there are different thresholds for troponin that are relevant for risk classification [10].

It is great that this safe and potentially cost-saving Dutch clinical product, the HEART score, is being accepted globally. Yet to be fully implemented we all have to accept the minor risk of missing an ACS as before, but thus far without increasing mortality.
